# Effect of Extraction Method on the Phenolic and Cyanogenic Glucoside Profile of Flaxseed Extracts and their Antioxidant Capacity

**DOI:** 10.1007/s11746-015-2729-x

**Published:** 2015-10-19

**Authors:** Katarzyna Waszkowiak, Anna Gliszczyńska-Świgło, Veronique Barthet, Joanna Skręty

**Affiliations:** Faculty of Food Science and Nutrition, Poznań University of Life Sciences, ul Wojska Polskiego 31, 60-624 Poznań, Poland; Faculty of Commodity Science, Poznań University of Economics, al Niepodległości 10, 61-875 Poznań, Poland; Grain Research Laboratory, Canadian Grain Commission, 1404-303 Main Street, Winnipeg, MB R3C 3G8 Canada

**Keywords:** Flaxseed, Solvent extraction, Phenolic compound, Cyanogenic glucoside, Antioxidant activity

## Abstract

The application of flaxseed extracts as food ingredients is a subject of interest to food technologists and nutritionists. Therefore, the influence of the extraction method on the content and composition of beneficial compounds as well as anti-nutrients is important. In the study, the effects of two solvent extraction methods, aqueous and 60 % ethanolic, on phenolic and cyanogenic glucoside profiles of flaxseed extract were determined and compared. The impact of extracted phenolic compounds on the antioxidant capacity of the extracts was also investigated. Defatted meals from brown and golden flax varieties were used as extraction material. The ethanolic extraction was more selective for phenolics (100.8–131.7 mg g^−1^) than the aqueous one (11.5–15.7 mg g^−1^). However, the contribution of particular phenolic compounds to total phenolics was much more dependent on flax variety than extraction method. A strong relationship was observed between both radical scavenging and ferric reducing activity and the content of phenolics (particularly secoisolariciresinol diglucoside). The correlation between extract chelating ability and phenolics was moderate suggesting that other flaxseed compounds are involved in this activity. The extraction method strongly affected cyanogenic glucoside content of flaxseed extracts; the aqueous extraction caused 96 % reduction in cyanogenic glucoside content (0.56–0.62 mmol g^−1^) when compared to the content in defatted meal (9.1–11.6 mmol g^−1^). On the contrary, ethanolic extraction resulted in the high cyanogenic glucoside content in the extracts (71–89 mmol g^−1^). The results reveals that ethanolic extraction gives extracts rich in antioxidant lignans; aqueous extracts have lower antioxidant activity than ethanolic but cyanogenic glucosides are significantly reduced.

## Introduction

Health benefits of flaxseed (*Linum usitatissimum* L) are well established [1]. They are mainly attributed to the biological activities of certain compounds, i.e. α-linolenic acid, dietary fibre, unique proteins, and phenolic compounds [2]. Currently, flax lignans are in focus (mainly secoisolariciresinol diglucoside, SDG) because of their estrogenic and antioxidant activity [3]. The SDG functions are related to crucial health benefits of flaxseed, i.e. anti-inflammatory activity, protection from certain type of tumours (mostly hormone-dependent carcinomas) and cardiovascular diseases, and even type-2 diabetes [1, 2, 4]. Flaxseed is the richest source of SDG. The content of SDG varies between 9 and 30 mg g^−1^ of defatted flaxseed meal [5]. In the seed, SDG is incorporated into large complexes; it is an ester-linked with 3-hydroxy-3-methylglutaric acid and other phenolics (e.g. hydroxycinnamic acid derivatives and flavonoid herbacetin diglucoside) [6]. It was found that the total content of phenolic acids and their derivatives in flaxseeds varied between 8 and 10 mg g^−1^ of seeds [7]. They seem to be a part of flaxseed antioxidant system because of their antioxidant activity which is related to the hydroxyl group in their ring [6, 8]. Flaxseed is also a source of valuable proteins and bioactive peptides that are potent functional food ingredients due to their well-established biological activity (e.g. antioxidant, anti-inflammatory, anti-hypertensive and anti-cholesterol properties) as well as food preservation capacity (anti-fungal activity) [9]. The functional properties of proteins can be considered in mixture with other flax bioactive compounds such as lignans and mucilage which are present in flaxseed meal and extracts.

Solvent extraction is the most common method to recover bioactive compounds from plant material. The extraction yield, and consequently the biological activity of plant extract, is strongly affected by the applied solvent [10]. Various solvents have been utilised for flax bioactive compound extraction: organic [10–12] and/or aqueous [13, 14]. It was shown [11] that the amount of antioxidant compounds (total phenolics and flavonoids) extracted from flaxseeds was affected by solvent polarity, i.e. a less polar solvent (ethyl acetate) was less efficient than a more polar one (70 % methanol). Water or ethanol and their mixtures are often recommended to prepare extracts because of their difference in polarity that could extract different compounds and their acceptability for human consumption [15]. Such extracts could be safely introduced into food products without risking an unacceptable level of hazardous solvent residues. Boussetta et al. [16] compared the total phenolics determined by Folin–Ciocalteu reagent and SDG content in the aqueous and ethanolic extracts obtained from HVED (high voltage electrical discharge) pre-treated flaxseeds. However, there is lack of comprehensive studies concerning the comparison of the extraction methods (aqueous vs. ethanolic) which include analysis of complex phenolic profiles showing both composition and content of particular phenolics after extraction, as well as their impact on antioxidant activity of the obtained extracts. The impact of flax variety on the phenolic profile of the extracts has also not been studied.

Flaxseed contains some anti-nutrients, e.g. cyanogenic glucosides. They are secondary plant metabolites which are composed of an ∝-hydroxynitrile aglycone and a sugar moiety [17]. After plant tissue damage (by chewing or technological processing), cyanogenic glucosides are converted to hydrogen cyanide (HCN) by a two-step process [17]: first, cyanogenic glucosides are decomposed to cyanohydrins (∝-hydroxynitriles) and sugars by β-glycosidase; next, cyanohydrins can decompose (spontaneously or in an enzymatic reaction catalysed by hydroxynitrile lyase) and form HCN. Hydrogen cyanide can also be released upon acid hydrolysis [18]. Both HCN and its anion form (CN^−^) are toxic to animal and human since their ability to link ions such as iron, copper or manganese; the ions are functional groups of enzymes, particularly those of the cytochrome respiratory chain. Exposure to cyanides may lead to acute, fatal intoxication. However, chronic intoxication has also been observed; it has been proved that long-term exposure to cyanide released from the rich-in-cyanogenic-plant diet is responsible for human central nervous syndrome (spastic paraparesis) called Konzo [19, 20]. The level of cyanogenic glucosides in flaxseed depends on the plant variety, climate, season, and soil type [21]. Official analysis of flaxseed cakes and meals in EU (Belgium) revealed up to 338 mg total cyanide equivalents per kilogram [17]. According to EFSA recommendation [17], the control of cyanogenic glucosides level in common food/feeding-stuff materials (e.g. flaxseed by-products and cassava chips) as well as processing impact on their content should be intensified. Considering toxicity of cyanides to human, the effect of extraction method on cyanogenic glucoside concentration in flaxseed extracts should also be monitored, particularly in the case of their application in food industry. Due to lack of data available to clarify the problem, it was decided to study how aqueous and ethanolic extractions affect the composition and content of cyanogenic glucosides in flaxseed extracts.

The aim of the study was to determine the effect of solvent extraction method (aqueous and 60 % ethanolic) on phenolic and cyanogenic glucoside profiles of flaxseed extracts (i.e. their composition and content). The water or 60 % ethanol solvents selected to prepare the extracts are considered as safe food additives. The impact of phenolic profile on the antioxidant capacity of the extracts (antiradical, reducing, and chelating ability) was also analysed. Three flaxseed varieties, both brown and golden seeds, were selected as the research material.

## Materials and Methods

### Standards and Reagents

2,2-Diphenyl-1-picryl-hydrazyl (DPPH), 6-hydroxy-2,5,7,8-tetramethylchroman-2-carboxylic acid (Trolox), di-potassium peroxodisulphate, 2,4,6-tripyridyl-s-triazine (TPTZ), iron (II) sulphate heptahydrate, iron (III) chloride hexahydrate, ferrozine, iron (II) chloride tetrahydrate, caffeic, *p*-coumaric and ferulic acids, methyl-α-d-glucopyranoside, phenyl-β-d-glucopyranoside and 1-methylimidazole were purchased from Sigma–Aldrich (USA), while 2,2ʹ-azino-bis(3-ethylbenzothiazoline-6-sulphonic acid) acid diammonium salt (ABTS) was obtained from Fluka (USA), and bistrimethylsilylacetamide and trimethylsilylchlorosilane from Regis Technologies (USA). Lignan standards were purchased from PhytoLab (Germany), and linustatin and neolinustatin from Chromadex (Santa Ana, CA, USA).

### Materials

The seeds of three Polish high-α-linolenate flax varieties (IHAR, Poland) were selected as research material: brown (Szafir variety) and two golden seeds (Oliwin and Jantarol varieties). Flaxseeds were milled using a ZM 200 mil ( 1 mm sieve; Retch, Germany). Defatted meals were prepared from milled flaxseeds (double cold extraction with hexane [22]) and utilised for aqueous and ethanolic extractions. The extracts were stored at 4 °C for further use.

### Extraction Methods

#### Aqueous Extraction

Aqueous extracts were prepared according to Waszkowiak and Rudzińska [22]. Briefly, 20 g of defatted flax meal was extracted with water (meal to water ratio of 1:15, m/v) under constant stirring with a magnetic stirrer at ambient temperature for 1 h. The supernatant was collected after centrifugation (25 min, 1500*g*; centrifuge 5702R; Eppendorf, Germany) and freeze-dried (Alpha 1-4 LSC Freeze dryer; Christ, Germany).

#### Ethanolic Extraction

Ethanolic extraction was carried out by the method of Waszkowiak et al. [23]. Defatted flax meal (20 g) was extracted twice with 60 % aqueous ethanol (meal to extraction solvent ratio of 1:7.5, m/v) under constant vigorous shaking (labscale orbital shaker GFL 3005; Germany) at ambient temperature for 1 h. The extract solution was separated from meal by filtration (Munktell & Filtrak filter paper no 1289) and centrifugation (20 min, 1500*g*). The supernatant was collected, evaporated using rotary vacuum evaporator (Buchi, Switzerland) to remove ethanol, and then freeze-dried.

The procedures were selected on the basis of the previously reported results regarding optimal conditions of aqueous and ethanolic extraction [15, 24, 25]. The extraction process was repeated four times. The extracts were pooled and mixed to obtain enough material for all experiments.

### Determination of Phenolic Compounds

Phenolic profiles of the flaxseed extracts were analysed according to the protocol described below.

#### Alkaline Hydrolysis

Solution of flaxseed extract (in water or 60 % ethanol, 0.200 g mL^−1^) was mixed with an equal volume of 2 mol L^−1^ sodium hydroxide. Hydrolysis was carried out for 2 h at room temperature. The reaction was stopped by addition of 36 % hydrochloric acid (1.2 mol L^−1^ final concentration in the sample). The sample was centrifuged (8000*g*, 5 min) and the supernatant was submitted to HPLC analysis.

#### Acid Hydrolysis

After alkaline hydrolysis, 1 mL of solution was taken and an equal volume of 4 mol L^−1^ hydrochloric acid was added. Hydrolysis was carried out for 1 h at 85 °C. The sample was then cooled and centrifuged (8000*g*, 5 min). The supernatant was submitted to HPLC analysis.

#### Qualitative and Quantitative Analysis of Phenolic Compounds

After hydrolysis (alkaline and acid), the main flax phenolic compounds, i.e. lignans (SDG and SECO; secoisolariciresinol), phenolic acids and their derivatives were identified and quantified by HPLC. All HPLC analyses were performed at room temperature on a Waters 600 high performance liquid chromatograph (Waters, Millford, MA, USA) equipped with a Cadenza 5CD-C18 column (4.6 × 75 mm, 5 µm; Imtakt, Japan). Injection volume was 10 μL. A mobile phase gradient with acetonitrile (solvent A) and 0.1 % trifluoroacetic acid (solvent B) was developed: linear increment from 12 to 80 % of solvent A in 14 min at a flow rate of 0.7 mL min^−1^ and followed by a decrease to 12 % acetonitrile in 1 min at a flow rate of 1.0 mL min^−1^ which was kept for an additional 10 min to re-equilibrate the column. In the next minute, the flow rate was decreased to 0.7 mL min^−1^.

The eluate was monitored using a photodiode-array 996 detector set at the wavelength characteristics of the tested compounds. The identification of phenolic compounds was done after alkaline and acid hydrolysis by comparing their retention times with those of corresponding standards. Moreover, a Waters 996 photodiode-array detector was used to identify the compounds on the basis of their absorption spectra. The compounds which standards are unavailable were identified based on the elution order of flaxseed phenolics and the formation or the decline under acid or alkaline hydrolysis. For details see [26]. Phenolic compounds were quantified using the external standard method. Each sample was injected at least three times and at least three independent samples were prepared for each extract.

### Determination of Protein Content

Protein content was determined in aqueous extracts by the Kjeldahl method [27] using a Kjeltec-2200 System (Tecator, Sweden).

### Determination of Cyanogenic Glucoside Content

In the extracts and defatted flaxseed meal (for comparison purposes), cyanogenic glycosides were determined using the GC method as described previously [28, 29] and analysed on an Agilent Technologies (Wilmington, DE, USA) 6890 gas chromatograph equipped with a flame ionisation detector and controlled by an Agilent Chemstation (A.09.03 build 1417). The column was a Supelco (St. Louis, MI, USA) SPB-17 column (30 m × 0.32 mm, 0.25 µm film thickness). The GC oven program was as follows: an initial temperature of 190 °C for 3 min, a 40 °C min^−1^ ramp to 280 °C, followed by a 4.75 min hold (total run time was 10 min). The hydrogen was used as the carrier gas at a flow of 2.5 mL min^−1^. Linustatin, neolinustatin, lotaustralin and linamarin were quantified using standard curves made of stock solutions of each standard at 0.500 mg mL^−1^. Internal standard solutions (methyl-α-d-glucopyranoside and phenyl-β-d-glucopyranoside) were also prepared at 0.500 mg mL^−1^.

### Antioxidant Capacity Analysis

Antiradical and ferric reducing power as well as Fe(II)-chelating ability were analysed to estimate the antioxidant capacity of flaxseed ethanolic and aqueous extracts. Each assay was performed at least three times (three independent analytical experiments) and at least three independent replicates were prepared for each standard and sample.

#### Antiradical Power—DPPH^•^ and ABTS^•+^ Assay

##### DPPH Assay

The DPPH^•^ assay was performed according to the Sharma and Bhat protocol [30] based on the scavenging of DPPH^•^ free radical at a concentration of 50 μmol L^−1^ in buffered methanol.

Stock solutions of the flaxseed extracts were prepared daily in buffered methanol (mixture of methanol and 100 mM acetic buffer, pH 5.57, in proportion of 3:2) and then diluted to obtain various solutions at concentrations within the assay activity range. The final ethanolic and aqueous extract concentrations were 0.02–0.20 and 0.20–1.20 g L^−1^, respectively. The results were expressed as the percentage of scavenging activity in comparison to the control sample (without extract); then log (concentration)-response curves were plotted. For comparison purposes and correlation analyses, EC50 value (i.e. effective extract concentration that reduces DPPH^*•*^ radicals by half) was calculated based on the linear regression equation.

##### ABTS Assay

The ABTS^•+^ radical cation decolourisation spectrophotometric assay in PBS (phosphate-buffered saline, pH 7.4) was carried out applying a method of Re et al. [31].

Stock solutions of the extracts were prepared daily (the aqueous extracts in PBS buffer and the ethanolic ones in 60 % ethanol). The solutions were diluted to obtain four suitable concentrations within the assay activity range. The final concentrations were 0.02–0.04 and 0.10–0.30 g L^−1^ for the ethanolic and the aqueous extracts, respectively. Standard Trolox solutions were prepared in the range of 0–20 μmol L^−1^ and the standard curve was plotted. The decrease (%) of absorbance at 734 nm was calculated and the results were expressed as mmol Trolox g^−1^.

#### Ferric Reducing Antioxidant Power—FRAP Assay

The FRAP assay is based on Fe(III) to Fe(II) reduction at pH 3.6 and formation of a blue ferrous–TPTZ (tripyridyltriazine) complex that is photometric detected at 593 nm. The reaction is non-specific and allows estimation of the total reducing power. In the study, the FRAP assay was performed according to Benzie and Strain protocol [32].

Stock solutions of the extracts were prepared daily (the aqueous extracts in deionised water and the ethanolic ones in 60 % ethanol). The stock solutions were diluted to obtain four appropriate concentrations within the assay activity range. The final ethanolic and aqueous extract concentrations were 0.03–0.06 and 0.30–0.60 g L^−1^, respectively. Iron (II) sulfate heptahydrate (FeSO_4_•7H_2_O) was utilised as a standard. Standard FeSO_4_ solutions ranging from 0.1 to 1.0 mmol L^−1^ were analysed (final concentration of FeSO_4_ in the assay ranged from 3.3 to 33.3 μmol L^−1^) and the standard curve was plotted. Ferric reducing activities of flax extracts were expressed as millimoles of FeSO_4_ per gram of extract.

#### Fe(II)-Chelating Assay

The Fe(II)-chelating activity of the flaxseed extracts was determined by a modified spectrophotometric method of Tang et al. [33].

The assay was performed with various solutions of flaxseed ethanolic (in 60 % ethanol; final concentrations: 0.05–0.20 g L^−1^) and aqueous extracts (in deionised water; final concentrations: 0.63–2.50 g L^−1^). The results were expressed as the chelating ability (%); then log (concentration)-response curves were plotted. For comparison purposes and correlation analyses, EC50 value [i.e. effective extract concentration that complexes half of Fe(II) ions] was calculated based on the linear regression equation.

### Statistical Analysis

Statistical analyses were conducted using STATISTICA (v.9.0; StatSoft). Data were expressed as mean ± standard deviation (SD) of three series (three independent samples) and three independent measurements for each sample (*n* = 9).

The effect of extract type on the antioxidant activity was analysed. Analysis of variance (ANOVA) for a CRD (completely randomised design) experiment was carried out and Tukey’s multiple range test at a significance level of *P* ≤ 0.05 was applied to compare the means. Moreover, relationships between variables (i.e. among antioxidant activities of flaxseed extracts and between antioxidant activities and content of phenolic compounds; *n* = 6) were examined by linear regression analyses and Pearson’s correlation coefficients (*r*) were calculated.

## Results and Discussion

### Effect of Extraction on the Composition and Content of Phenolics

In the present study, water or 60 % ethanol was used to prepare extracts that could be safely introduced into food products. The analysis of phenolic profile of the tested extracts (i.e. the composition and content, Table 1) showed that secoisolariciresinol diglucoside (SDG) was the main phenolic compound of all flaxseed extracts after alkaline hydrolysis. Ferulic and *p*-coumaric acid as well as phenolic acid glucosides (i.e. *p*-coumaric, caffeic and ferulic acid) were also found in the extracts after the hydrolysis. The main compounds after alkaline-acid hydrolysis were: secoisolariciresinol (SECO), ferulic acid and *p*-coumaric acid. Li et al. [26] proposed the hydrolytic reaction pathway of SDG oligomers in a reaction medium containing methanol or ethanol. According to this pathway, SDG is released by the alkaline hydrolysis and then deglucosilates into SECO under acidic conditions. *p*-coumaric acid glucoside and ferulic acid glucoside released from the SDG oligomers under alkaline conditions are subsequently esterified by alcohol under acid conditions (hydrochloric acid treatment). This explains the presence of compounds tentatively identified as esters of both *p*-coumaric and ferulic acids in the ethanolic but not in the aqueous extracts.

**Table 1:** Content of phenolic compounds (mg g^−1^) in flaxseed extracts

Phenolic compounds	Ethanolic flaxseed extracts			Aqueous flaxseed extracts		
	Brown Szafir var.	Golden Oliwin var.	Golden Jantarol var.	Brown Szafir var.	Golden Oliwin var.	Golden Jantarol var.
SDG^a^	107.37 ± 3.00	77.96 ± 1.00	94.02 ± 0.95	9.55 ± 0.13	10.80 ± 0.13	14.52 ± 0.22
SECO^b^	21.74 ± 0.60	15.85 ± 0.20	19.06 ± 0.19	2.11 ± 0.03	2.36 ± 0.03	3.11 ± 0.04
*p*-Coumaric acid glucoside^a,c,e^	9.85 ± 0.14	9.77 ± 0.11	2.46 ± 0.03	0.73 ± 0.04	1.68 ± 0.07	0.36 ± 0.01
Caffeic acid glucoside^a,c,f^	2.18 ± 0.04	2.01 ± 0.01	1.26 ± 0.14	0.17 ± 0.01	0.23 ± 0.01	0.21 ± 0.00
Ferulic acid glucoside^a,c,g^	1.92 ± 0.21	1.82 ± 0.02	1.05 ± 0.01	0.14 ± 0.01	0.20 ± 0.01	0.20 ± 0.00
*p*-Coumaric acid^a^	1.30 ± 0.02	1.26 ± 0.02	0.34 ± 0.00	0.16 ± 0.01	0.19 ± 0.01	0.09 ± 0.01
*p*-Coumaric acid^b^	7.33 ± 0.09	7.78 ± 0.24	2.05 ± 0.05	0.51 ± 0.02	0.68 ± 0.04	0.42 ± 0.04
Ferulic acid^a^	9.04 ± 0.24	13.69 ± 0.14	1.71 ± 0.04	0.76 ± 0.04	1.88 ± 0.05	0.37 ± 0.01
Ferulic acid^b^	10.64 ± 0.12	14.45 ± 0.14	3.17 ± 0.05	0.87 ± 0.01	1.81 ± 0.05	0.56 ± 0.06
Caffeic acid^b^	2.77 ± 0.03	1.29 ± 0.02	0.61 ± 0.02	0.12 ± 0.01	0.14 ± 0.00	0.08 ± 0.01
*p*-Hydroxybenzoic acid^b^	1.60 ± 0.01	0.77 ± 0.03	0.38 ± 0.01	0.13 ± 0.01	0.14 ± 0.01	0.17 ± 0.01
*p*-Coumaric acid ester^b,d,e^	0.90 ± 0.03	0.69 ± 0.12	0.25 ± 0.01	ND	ND	ND
Ferulic acid ester^b,d,g^	3.83 ± 0.42	1.80 ± 0.41	0.39 ± 0.03	ND	ND	ND
Total after alkaline hydrolysis^a^	131.65 ± 2.88	106.51 ± 1.11	100.83 ± 0.96	11.50 ± 0.21	14.98 ± 0.17	15.73 ± 0.23
Total after alkaline-acid hydrolysis^b^	48.80 ± 0.86	42.63 ± 0.80	25.92 ± 0.33	3.73 ± 0.05	5.13 ± 0.07	4.34 ± 0.12

Mean (*n* = 9) ± SD

*SDG* secoisolariciresinol diglucoside, *SECO* secoisolariciresinol, *ND* not detected

^a^Present after alkaline hydrolysis of extract solution

^b^Present after alkaline and acid hydrolyses of extract solution

^c^Identified based on elution order of flax phenolics and its decline after acid hydrolysis; for details see [26]

^d^Identified based on elution order of flax phenolics and formation conditions; for details see [26]

^e^Quantified as *p*-coumaric acid

^f^Quantified as caffeic acid

^g^Quantified as ferulic acid

The flaxseed ethanolic extracts contained from 6 to 11 times more total phenolics (TP, after alkaline hydrolysis) and SDG than the aqueous extracts in the case of all tested varieties (golden and brown seeds). TP content (Table 1) ranged from 100.8 to 131.6 mg g^−1^ in the ethanolic extracts and from 11.5 to 15.7 mg g^−1^ in the aqueous one; SDG contents were 94.0–107.4 and 9.5–14.5 mg g^−1^, respectively. This shows that the extraction method greatly affected the content of phenolics in the extracts; the ethanolic extraction was more selective for phenolic compounds than the aqueous one. The results can be explained by difference in polarity of the extraction solvents. Zhang et al. [15] reported that extraction yield of flaxseed lignans increased when the mixture of ethanol and water was applied instead of pure ethanol. They found that the best ethanol concentration was in the range of 56–83 %; however, the extraction yield decreased when the water content in extraction solvent was 50 %. Our preliminary study concerning the effect of ethanol concentration in extraction solvent on phenolic content and antioxidant activity of flaxseed extracts indicated 60 % ethanol as the best for extraction; we found that 60 and 65 % ethanolic extracts showed the highest antiradical activity (DPPH^•^ assay), and that the 60 % one had significantly higher phenolic compound content (both SDG and total phenolics) than the other extracts (unpublished data).

The results of this study showed that the content of phenolic compounds varied in flaxseed ethanolic or aqueous extracts depending on extraction material, i.e. flax variety. Among the ethanolic extracts, the highest level of TP and SDG (after alkaline hydrolysis) was observed for the brown seed (Szafir var.) extract (Table 1). The lowest SDG content was found in the ethanolic extract of golden Oliwin var., whereas the lowest content of phenolic acids and their derivatives was detected in the golden Jantarol extract. Among the aqueous extracts, the one from golden Jantarol var. was the richest in TP and SDG, whereas from the brown Szafir var. this was the poorest.

**Fig. 1:**
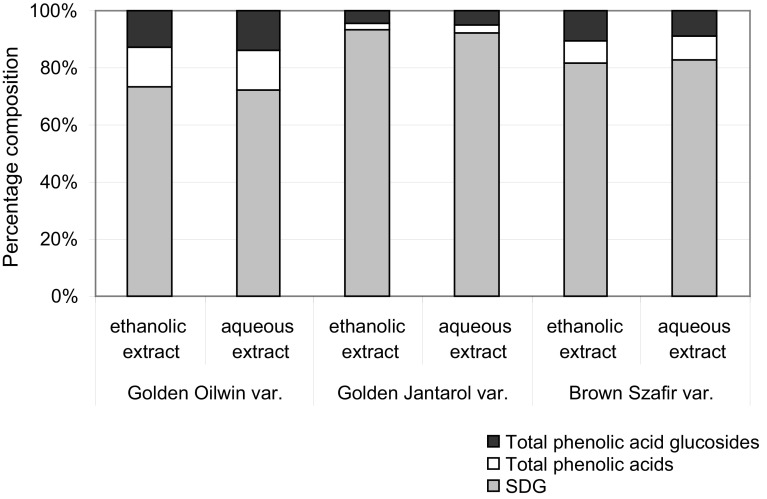
Percentage contribution of SDG lignan, phenolic acids and their glucoside derivatives (amount after alkaline hydrolysis) to total phenolics in the ethanolic and aqueous extracts

The percentage contribution of SDG lignan, phenolic acids and their glucoside derivatives (amount after alkaline hydrolysis) to TP in the ethanolic and aqueous extracts from Oliwin, Jantarol and Szafir var. are presented in Fig. 1. It was observed that the contribution of particular phenolic compounds to TP content was similar for both ethanolic and aqueous extracts obtained from the same variety. This suggested that the composition of extracted phenolics is much more dependent on flax variety (i.e. raw material composition) than extraction method. Therefore, a careful selection of extraction material seems to be one of the crucial factors which should be considered in industrial production of flaxseed extracts. Further research should be performed to elucidate this finding.

### Impact of Phenolic Profile on Flaxseed Extract Antioxidant Capacity

In the study, antioxidant capacity of ethanolic and aqueous extracts from the seeds of three flax varieties was estimated and compared. The radical scavenging activity (DPPH^•^ and ABTS^•+^), ferric reducing power (FRAP), and Fe(II)-chelating ability were analysed (Table 2).

**Table 2:** Antioxidant capacity of ethanolic and aqueous flaxseed extracts

Antioxidant activity	Flaxseed variety		
	Brown Szafir var.	Golden Oliwin var.	Golden Jantarol var.
Ethanolic flaxseed extracts			
Antiradical activity			
DPPH^•^assay—EC50 (g L^−1^)	0.06 ± 0.01^b^	0.08 ± 0.01^a^	0.08 ± 0.01^a^
ABTS^•+^ assay (mmol Trolox g^−1^)	5.06 ± 0.28^c^	4.30 ± 0.17^b^	3.83 ± 0.17^a^
Reducing activity			
FRAP assay (mmol FeS0_4_ g^−1^)	4.96 ± 0.19^c^	3.80 ± 0.23^a^	4.37 ± 0.11^b^
Chelating ability—EC50 (g L^−1^)	0.12 ± 0.01^a^	0.13 ± 0.01^a^	0.11 ± 0.01^a^
Aqueous flaxseed extracts			
Antiradical activity			
DPPH^•^ assay—EC50 (g L^−1^)	0.51 ± 0.02^a^	0.43 ± 0.01^b^	0.39 ± 0.02^b^
ABTS^•+^ assay (mmol Trolox g^−1^)	0.54 ± 0.08^b^	0.46 ± 0.04^a^	0.50 ± 0.08^ab^
Reducing activity			
FRAP assay (mmol FeS0_4_ g^−1^)	0.60 ± 0.01^b^	0.44 ± 0.01^a^	0.61 ± 0.01^b^
Chelating ability—EC50 (g L^−1^)	2.86 ± 0.18^c^	4.28 ± 0.67^b^	6.54 ± 0.01^a^

*EC50* The effective extract concentration needed to reduce the initial DPPH^•^ amount by half (DPPH^•^ assay) or complex half of Fe(II) ions (chelating ability)

Mean (*n* = 9) ± SD; means with different letters in the same row were significantly different (one-way ANOVA and Tukey’s test, *P* < 0.05; *a* is the lowest activity)

A relationship (*P* < 0.01) between extract concentration and percentage of DPPH^•^ scavenging was found (results not shown). The EC50 values were calculated to study the effect of variety and extraction method on the extract antiradical activity (Table 2). The lower EC50 value means the higher DPPH^•^ radical scavenging activity of an extract, since a lower extract concentration was needed to reduce the initial DPPH^•^ amount by half. It was found that ethanolic extracts showed higher antiradical activity than aqueous one. The calculated EC50 values showed that about 5–8.5 times higher concentration of aqueous extract was needed to neutralise half of DPPH^•^ radicals as compared to ethanolic extract obtained from the same flax variety. Similar trend was observed with the ABTS^•+^ assay results; the activity of ethanolic extract was 7.5–9 times higher than the aqueous one (Table 2).

In the case of both DPPH^•^ and ABTS^•+^ scavenging assays, the highest activity was found for the ethanolic extract from brown seed variety (Szafir var.). Its highest activity corresponded with the highest content of TP and SDG (Table 1). Among aqueous extracts, the effect of variety on antiradical activity was different; the extract of brown seed variety showed lower activity in DPPH^•^ assay and higher in ABTS^•+^ assay as compared to those of the golden seed varieties (Oliwin and Jantarol var.).

The results of FRAP assay showed that the relationships between extraction method (applied solvent) or material (flax variety) and extract ferric reducing activity were similar to those described above (Table 2). The reducing activity of the ethanolic extract was noted to be about 7–9 times higher than the activity of aqueous extract obtained from the same flaxseed variety. Among ethanolic extracts, the strongest ferric reducing ability was observed for the extract of brown seeds (Szafir var.). The weakest activities were found for the ethanolic and aqueous extracts of golden Oliwin seeds.

A strong relationship between the results of antiradical assays (DPPH^•^ and ABTS^•+^; *r* = −0.969) and FRAP assay was established (Table 3). High correlations were found between ABTS^•+^ and FRAP assay results (*r* = 0.988), as well as DPPH^•^ (EC50) and FRAP assay results (*r* = −0.970). Anwar and Przybylski [10] also reported high correlation between antiradical activity (DPPH^•^ assay) and ferric reducing power (FRAP) for alcoholic flax extracts.

**Table 3:** Correlations among antioxidant activities of flaxseed extracts (ethanolic and aqueous) and between the antioxidant activities and the content of SDG or total phenolics

Factors	Correlation coefficient *r*			
	DPPH^•^ assay—EC50 (g L^−1^)	ABTS^•+^ assay (mmol Trolox g^−1^)	FRAP assay (mmol FeS0_4_ g^−1^)	Chelating ability—EC50 (g L^−1^)
DPPH^•^ assay—EC50 (g L^−1^)	–	−0.969**	−0.970**	NS
ABTS^•+^ assay (mmol Trolox g^−1^)		–	0.988***	−0.888*
FRAP assay (mmol FeS0_4_ g^−1^)			–	−0.885*
Chelating ability—EC50 (g L^−1^)				–
SDG (mg g^−1^)	−0.972**	0.983***	0.998***	−0.866*
TP (mg g^−1^)	−0.974**	0.998***	0.992***	−0.874*

*SDG* secoisolariciresinol diglucoside, *TP* total phenolics, *EC50* as in Table 2

Linear regression: *y* = *ax* + *b*; ***P < 0.001, **P < 0.01, *P < 0.05, *NS* the finding is not statistically significant (*P* ≥ 0.05)

The results of the present study showed that the flaxseed extracts had Fe(II)-chelating ability and that it was concentration-dependent (results not shown). A relationship (*P* < 0.01) was found between extract concentration and percentage of Fe(II) chelation. The EC50 value was calculated to study the effect of variety and extraction method on the Fe(II)-chelating ability of the flaxseed extracts (Table 2). It was found that the aqueous extract had lower Fe(II)-chelating ability than ethanolic one obtained from the same flaxseed variety. The chelating abilities of the ethanolic extracts were similar, irrespective of the flax variety. Among aqueous extracts, the extract from brown seed variety showed significantly higher chelating ability (the lowest EC50 value) comparing to those from both golden varieties. The moderate relationships between the Fe(II)-chelating ability of flaxseed extracts and FRAP or ABTS^•+^ results were established and no significant relationship (*P* > 0.05) between the chelating ability and DPPH^•^ radical scavenging activity was found (Table 3).

The differences in antioxidant activity of the flaxseed ethanolic and aqueous extracts corresponded to the differences in the content of phenolics described above. Statistical analysis was performed to confirm the findings and a linear relationship between the phenolic compound content and the antioxidant activity of flaxseed extracts was found (Table 3). Strong relationships between the results of both antiradical (DPPH^•^ and ABTS^•+^) and ferric-reducing ability (FRAP) and TP as well as SDG content were established (*r* = 0.972–0.998). The relationship between the chelating ability and TP content was moderate (*r* = −0.874). High correlation between the total content of phenolics (estimated with Folin–Ciocalteu reagent) and antioxidant activity of flax extracts has been previously reported [10–12].

The results of the study showed that SDG lignan is an important element of the flaxseed extract antioxidant system. It was previously reported that SDG acted as a direct radical scavenger [34] and lipid peroxidation inhibitor [35]. Hu et al. [36] found that SDG was effective against DPPH^•^ and 2,2ʹ-azobis(2-amidinopropane) dihydrochloride (APPH) radicals at dietary equivalent concentrations (i.e. 25–200 and 10–100 μmol of SDG, respectively). Flax lignans also showed a high antioxidant activity in the FRAP assay [37]. The 4-hydroxy-3-methoxy aromatic structure of SDG was suggested to be responsible for its antioxidant activity [36–38]. The antioxidant capacity was also exhibited by SECO and natural phenolic oligomers extracted from flaxseeds [39].

However, the lower correlation coefficient between the iron chelating activity and the content of phenolics may suggest that other flaxseed compounds are involved in this activity. The assumption is supported by the results of Fucassi et al. [40]; they studied the affinity of SDG for metal cations and found a low value for Fe(II) as compared to plant phenolics with similar structural moieties. For aqueous extracts, some flaxseed proteins can be suspected of being involved in the chelating activity. The results of the present study showed that aqueous extracts obtained from the tested flaxseed varieties varied in total protein content. However, the amino acid profiles were similar for all extracts (data not presented). The highest protein content (per 100 g) was found in the extract from brown seed variety (42.2 ± 0.2 g) as compared with those from the golden seed varieties (about 35.8 ± 0.4 and 33.8 ± 0.4 g for Oliwin and Jantarol extracts, respectively). As it was described above, significantly higher chelating ability was observed for the brown seed extract with the highest protein content. Further research should be performed to elucidate this finding.

### Effect of Extraction on Cyanogenic Glucoside Composition and Content

The effect of extraction method (60 % ethanol or aqueous) on cyanogenic glycoside content in the extracts was also studied since these compounds release toxic hydrogen cyanide upon hydrolysis (Table 4). Monitoring of cyanogenic glycoside content in the flaxseed extracts is particularly important when they are intended to be applied as food additives. The contents of cyanogenic diglucosides (linustatin and neolinustatin) and monoglucosides (linamarin and lotaustralin) in extracts and extracted material (defatted flax meals) were analysed. Based on the total cyanogenic glucoside (TCG) content, the hydrogen cyanide (HCN) equivalent was calculated. It represents the amount of cyanide (calculated as HCN) that the sample can release upon total degradation of all its cyanogenic constituents. The TCG content in the flaxseed ethanolic and aqueous extracts were also expressed per gram of defatted flax meal to calculate the TCG yield and to show the effect of extraction method on the content of these compounds in the extracts.

**Table 4:** Cyanogenic glucoside content in defatted flax meal and flaxseed extracts

Flax variety	Cyanogenic glucoside content (μmol g^−1^) and (mg∙100 g^−1^) in parentheses^a^				Total content (μmol g^−1^)^a^	HCN (mg kg^−1^)	Ratio Lin:Neo	TCG yield^b^
	Linamarin	Lotaustralin	Linustatin	Neolinustatin				
Defatted flaxseed meal								
Brown Szafir var.	0.15 ± 0.02 (3.6 ± 0.5)^c^	0.14 ± 0.01 (3.8 ± 0.3)	7.14 ± 0.47 (290.5 ± 19.4)	3.71 ± 0.25 (157.0 ± 10.4)	11.09 ± 0.53	300 ± 14	1.9	100 %
Golden Oliwin var.	0.16 ± 0.01 (3.9 ± 0.3)	0.15 ± 0.01 (3.4 ± 0.2)	7.81 ± 0.07 (319.7 ± 2.7)	3.47 ± 0.04 (147.0 ± 1.7)	11.59 ± 0.08	313 ± 2	2.2	100 %
Golden Jantarol var.	0.13 ± 0.01 (3.2 ± 0.3)	0.16 ± 0.00 (4.1 ± 0.0)	6.46 ± 0.30 (264.4 ± 12.4)	2.37 ± 0.13 (100.3 ± 5.4)	9.11 ± 0.33	246 ± 9	2.7	100 %
Aqueous flaxseed extracts								
Brown Szafir var.	0.03 ± 0.01 (0.7 ± 0.1)	0.12 ± 0.01 (3.2 ± 0.3)	0.36 ± 0.03 (14.9 ± 1.3)	0.09 ± 0.00 (3.6 ± 0.0)	0.60 ± 0.03	16 ± 1	4.2	2.3 % (0.25 ± 0.01)
Golden Oliwin var.	ND	0.06 ± 0.01 (1.7 ± 0.2)	0.47 ± 0.02 (19.2 ± 0.9)	0.09 ± 0.00 (3.6 ± 0.0)	0.62 ± 0.02	17 ± 1	5.5	3.8 % (0.44 ± 0.02)
Golden Jantarol var.	ND	0.06 ± 0.00 (1.6 ± 0.1)	0.41 ± 0.02 (16.8 ± 1.0)	0.09 ± 0.00 (3.6 ± 0.0)	0.56 ± 0.02	15 ± 1	4.8	4.3 % (0.39 ± 0.01)
Ethanolic flaxseed extracts								
Brown Szafir var.	7.88 ± 0.07 (194.8 ± 1.6)	2.96 ± 0.09 (77.3 ± 2.4)	51.54 ± 0.28 (2110.1 ± 11.3)	27.45 ± 0.13 (1162.4 ± 5.4)	89.83 ± 0.33	2428 ± 9	1.9	69.8 % (7.74 ± 0.05)
Golden Oliwin var.	17.44 ± 0.32 (431.2 ± 7.9)	6.19 ± 0.12 (161.7 ± 3.0)	37.82 ± 0.67 (1548.4 ± 27.6)	17.77 ± 0.17 (752.6 ± 7.0)	79.23 ± 0.77	2141 ± 21	2.1	66.4 % (7.70 ± 0.09)
Golden Jantarol var.	12.20 ± 0.24 (301.7 ± 6.0)	3.17 ± 0.07 (82.8 ± 1.7)	40.80 ± 0.64 (1670.3 ± 26.2)	15.07 ± 0.19 (638.2 ± 8.2)	71.25 ± 0.71	1926 ± 19	2.7	69.8 % (6.35 ± 0.10)

*ND* not detected, *HCN* hydrogen cyanide equivalent

^a^Contents in μmol g^−1^ of defatted meals or extracts, respectively

^b^ *TCG yield* TCG content (μmol g^−1^) in the flaxseed extract was calculated per gram of defatted flax meal (in parenthesis) and expressed as % of the TCG content in defatted meal

^c^Mean (*n* = 9) ± SD

The main cyanogenic glucoside identified in the tested materials was the cyanogenic diglucoside linustatin; neolinustatin, the other cyanogenic diglucoside, was also present (Table 4). Previous studies indicated that the cyanogenic diglucosides were present in mature flaxseeds and meals while cyanogenic monoglucosides were mostly detected in seedlings, flowers and immature seeds [21, 29]. The TCG content in flaxseed meals ranged from 9.11 to 11.59 μmol g^−1^; the lowest content was found in the golden Jantarol var. and the highest in the brown Szafir var. Oomah et al. [21] reported that cultivars affected cyanogenic glucoside content in flaxseeds.

The extraction solvent (water or 60 % ethanol) significantly affected the content of cyanogenic glucosides in the flaxseed extracts. The TCG contents in the extracts were much lower after aqueous than ethanolic extraction (Table 4). After aqueous extraction, the TCG content dropped by 96–98 % as compared to their initial content in the defatted flaxseed meals. Therefore, the content in aqueous extracts ranged from 0.56 to 0.62 μmol g^−1^ of extract. The extraction also changed the proportion of particular cyanogenic glucosides. The most significant changes were observed for neolinustatin and linustatin. Having estimated the linustatin to neolinustatin ratio, it was found that this ratio increased almost by two in aqueous extracts as compared to the ratio for the defatted flax meals (Table 4).

The present study showed that the aqueous extraction was promising method enabling extraction of valuable flaxseed constituents at the same time reducing levels of cyanogenic glucosides (anti-nutrients). Both aspects are important when the flaxseed extracts are intended to be utilised as diet supplements or food additives. The decrease in the cyanogenic glucoside content was likely the effect of the poor extractability of these compounds by water and flaxseed enzyme activation (also those involved in cyanogenic glucoside degradation). The results of Feng et al. [41] and Yang et al. [42] indicated that flaxseed processing with water (e.g. water-soaking or wet autoclaving) significantly reduced the cyanogenic glucoside amount.

On the contrary, ethanolic extraction resulted in a high TCG content in the extracts (Table 4). The TCG content ranged from 71 to 89 μmol g^−1^ for ethanolic extracts from golden Jantarol and brown Szafir var., respectively. It was found that 60 % ethanol was able to extract up to 70 % of cyanogenic glucosides from defatted flax meal (calculated TCG yield). It is likely that the applied ethanolic extraction is suitable to extract and concentrate both phenolics (mainly SDG) and cyanogenic glycosides, resulting in higher levels of these compounds in ethanolic extracts. Barthet and Bacala [29] reported that 70–80 % methanol was the most efficient for extraction of cyanogenic glycosides (linustatin and neolinustatin) from flaxseed.

It should be mentioned that despite of the higher cyanogenic glucoside content in the ethanolic extracts, they could still be utilised in food production because of their low addition level as functional food ingredient. Usually, plant ethanolic extract addition is below 0.1 %. The results of our previous study [23] indicated that the flaxseed extracts (0.05 % addition) can be utilised to prolong the shelf-life of meat products by protecting them from lipid oxidation and deterioration of their nutritional quality. Food fortification with a flaxseed ethanolic extract at the same level (0.05 %) will introduce about 0.12 mg HCN into product (100 g). This amount is much lower than the estimated acute toxic dose of HCN for adults (50 mg) [2] or the limit established by FAO [43] for cassava flour (10 mg kg^−1^). However, our present study indicates that the content of cyanogenic glucosides should be monitored in flaxseed ethanolic extracts, particularly in the case of diet/food fortification with the extracts since the extraction process concentrate these anti-nutrients. It may help to select a safe extract addition as well as to control the exposure to cyanide from the human diet.

## Conclusions

The results of the study show the effect of extraction method (solvent choice) and raw material (flax variety) on the phenolic profile as well as cyanogenic glucoside content in flaxseed extracts. Flaxseed extracts, particularly ethanolic one, showed antioxidant activity in the selected assays, i.e. antiradical, reducing, and Fe(II)–chelating ability. Strong linear relationships between the content of phenolics (particularly SDG) and the antiradical activity against DPPH^•^ radical, ABTS^•+^ radical cation, and the reducing power were established.

The results revealed that ethanolic extraction, more selective for phenolics than aqueous, can be a suitable method to obtain flaxseed extracts exhibiting considerable antioxidant activity. The ethanolic extracts could replace synthetic antioxidants to maintain food oxidative stability. However, cyanogenic glucoside contents in the extracts should be monitored, since their concentrations are higher after ethanolic than aqueous extraction. The present study also showed that aqueous extraction is an alternative to obtain flaxseed extracts having antioxidant activity and low cyanogenic glucoside content.
